# Evidence for habitual climbing in a Pleistocene hominin in South Africa

**DOI:** 10.1073/pnas.1914481117

**Published:** 2020-03-30

**Authors:** Leoni Georgiou, Christopher J. Dunmore, Ameline Bardo, Laura T. Buck, Jean-Jacques Hublin, Dieter H. Pahr, Dominic Stratford, Alexander Synek, Tracy L. Kivell, Matthew M. Skinner

**Affiliations:** ^a^Skeletal Biology Research Centre, School of Anthropology and Conservation, University of Kent, Canterbury CT2 7NR, United Kingdom;; ^b^Research Centre for Biological Anthropology, Liverpool John Moores University, Liverpool L3 3AF, United Kingdom;; ^c^Department of Human Evolution, Max Planck Institute for Evolutionary Anthropology, 04103 Leipzig, Germany;; ^d^Collège de France, 75231 Paris, France;; ^e^Institute for Lightweight Design and Structural Biomechanics, Vienna University of Technology, A-1060 Vienna, Austria;; ^f^Department of Anatomy and Biomechanics, Karl Landsteiner Private University of Health Sciences, A-3500 Krems an der Donau, Austria;; ^g^School of Geography, Archaeology and Environmental Studies, University of the Witwatersrand, 2000 Johannesburg, South Africa;; ^h^Evolutionary Studies Institute, University of Witwatersrand, 2050 Johannesburg, South Africa

**Keywords:** anthropology, human evolution, trabecular bone

## Abstract

Bipedalism is a defining trait of the hominin lineage, associated with a transition from a more arboreal to a more terrestrial environment. While there is debate about when modern human-like bipedalism first appeared in hominins, all known South African hominins show morphological adaptations to bipedalism, suggesting that this was their predominant mode of locomotion. Here we present evidence that hominins preserved in the Sterkfontein Caves practiced two different locomotor repertoires. The trabecular structure of a proximal femur (StW 522) attributed to *Australopithecus africanus* exhibits a modern human-like bipedal locomotor pattern, while that of a geologically younger specimen (StW 311) attributed to either *Homo* sp. or *Paranthropus robustus* exhibits a pattern more similar to nonhuman apes, potentially suggesting regular bouts of both climbing and terrestrial bipedalism. Our results demonstrate distinct morphological differences, linked to behavioral differences between *Australopithecus* and later hominins in South Africa and contribute to the increasing evidence of locomotor diversity within the hominin clade.

## Significance

Here we present evidence of hominin locomotor behavior from the trabecular bone of the femur. We show evidence for habitual use of highly flexed hip postures, which could potentially indicate regular climbing in a South African hominin from Sterkfontein, which is either *Paranthropus robustus* or *Homo*. Second, we present evidence that *Australopithecus africanus* likely did not climb at the frequencies seen in extant nonhuman apes, and exhibits a modern, human-like pattern of loading at the hip joint. These results challenge the prevailing view of a single transition to bipedalism within the hominin clade by providing evidence of climbing in a more recent, non-*Australopithecus* South African hominin, and add to the increasing evidence for locomotor diversity in the hominin clade.

Skeletal adaptations for bipedal locomotion in the hominin lineage date back to at least six million years ago (1). These bipedal adaptations are found throughout the skeleton, but those of the hip and knee are particularly important as these joints are central in determining how load is transferred through the lower limb. In modern humans, femoral adaptations for bipedalism include a relatively large femoral head, long neck (2), and a high bicondylar angle compared with extant apes, as well as flat, ellipsoid distal condyles and an elevated patellar lip (3, 4). Conversely, in African apes the femoral head is relatively small and the neck short (2), while the distal condyles are more circular (3, 4). Identifying bipedal adaptations in fossil apes is critical to placing them on the hominin lineage; however, the presence of such adaptations in the earliest fossil hominins (e.g., *Sahelanthropus* and *Orrorin*) is controversial (1, 5, 6). Generally accepted evidence for obligate bipedalism is found in later hominins, such as the australopiths (7–9). Here we test for evidence of committed terrestrial bipedalism and/or evidence for significant bouts of climbing in South African hominins, including *Australopithecus africanus*.

Adaptations for bipedalism appear in the tibia of the earliest known australopith, *Australopithecus anamensis* (9), however the absence of additional lower limb postcranial remains belonging to this taxon limits the interpretation of its locomotion. The more complete fossil record for *Australopithecus afarensis* includes femoral specimens with a long femoral neck and human-like femoral muscular organization in the proximal femur (10) as well as a raised patellar lip, ellipsoid condyles, and a deep patellar groove in the distal femur (4), suggesting that they frequently adopted bipedality. Similar traits are found in *Australopithecus africanus* (3). Furthermore, other South African fossils, including *Australopithecus sediba* (11) and *Australopithecus* sp. Sterkfontein Wits (StW) 573 (12, 13), strengthen this notion that australopiths were committed terrestrial bipeds. However, the different mosaics of human- and ape-like external traits in australopiths have led to debate over the form of bipedalism (14, 15), as well as the levels of arboreality in these taxa (16, 17). More definitive traits for mechanically modern human-like, obligate bipedalism appear in *Homo erectus* and most later *Homo* taxa (18–20), but the locomotion of other *Homo* taxa, including *Homo habilis*, is still poorly understood (21, 22).

Most studies of fossil hominin bipedalism have focused on external morphological traits (1, 4, 13). However, debates about behavioral interpretations based on external morphology have arisen due to the suggestion that, in the absence of strong selective pressure, primitive traits can be retained that are no longer functionally relevant (15). Additionally, it has been argued that some Pliocene hominins may exhibit functional divergence of the upper and lower limbs associated with selection for both arboreality and terrestrial bipedalism, respectively (13, 23).The discoveries of StW 573 (nicknamed “Little Foot”) (12, 13), *A. sediba* (24), *Homo floresiensis* (25), and *Homo naledi* (26) reveal additional unexpected combinations of ape-like and human-like morphologies in the hominin fossil record. To better understand actual, rather than potential, behavior in the past, this study focuses on reconstructing predominant joint positions habitually practiced by fossil hominin individuals through the analysis of internal bone structure (trabecular or cancellous bone) to clarify the locomotor repertoire in different species.

Investigation of trabecular architecture in long bones has proven integral in reconstructing behaviors in both extant and fossil humans, as well as other primates (27–31). This is because trabecular bone responds to load via modeling and remodeling, mainly altering the orientation of its struts, as well as the distribution and volume of bone (32). Analysis of trabecular architecture has revealed behavioral signals in the femoral head of primates (29, 30, 33). Our previous work has shown that within the femoral head, trabecular bone distribution differs between humans, African apes, and orangutans (30) and correlates with predicted pressure from habitual postures. Furthermore, within the femoral head, modern humans have highly aligned struts (expressed as high degree of anisotropy [DA]) and distinct strut orientation compared to other apes (29), traits that are consistent with obligate bipedalism. Bone volume fraction (expressed as bone volume/total volume [BV/TV]) is significantly lower in modern humans relative to great apes, but varies with activity levels, with more sedentary modern humans showing lower bone volume within the femoral head than more active humans (31). Trabecular studies in the femoral head (29) and distal tibia (27) of *A. africanus* have shown that the trabeculae are highly aligned and oriented in a similar manner to humans and distinct from chimpanzees. However, these studies focused on subvolumes (or two-dimensional [2D] slices) of trabecular bone and since trabecular structure is not homogeneously distributed across epiphyses (34), analyzing isolated volumes may obscure or limit functional interpretations. In particular, recent studies have shown that the analysis of subchondral trabecular bone distribution and architecture is crucial to revealing differences in joint loading across primates that practice different locomotor repertoires (27, 29, 34).

Here we conduct a comparative analysis of the three-dimensional (3D) trabecular bone distribution beneath the subchondral layer of the proximal femoral head in humans, other great apes and two fossil hominin specimens from the Sterkfontein Caves, South Africa: StW 311 and StW 522 (*SI Appendix*, Table S4). StW 522 derives from Member 4 which has been dated broadly to 2.8 to 2.0 Ma (35) (*SI Appendix*). This specimen has been attributed to *A. africanus* (36). The StW 311 proximal femur derives from the stratigraphically complex eastern end of Member 5 at Sterkfontein (named Member 5 East [M5E]) (37), where two infills are recognized, both of which are artifact and hominin bearing. The lower infill unit, recently dated to 2.18 Ma (38), contains *Paranthropus robustus* remains and Oldowan artifacts. However, previously it has been suggested to date from 1.7 to 1.4 Ma (37) and 1.4 to 1.2 Ma (35). The upper unit of M5E, dated to 1.7 to 1.4 Ma (37) or 1.3 to 1.1 Ma (35), is characterized by early Acheulean stone tools. Although StW 311 has been previously attributed to *A. africanus* (2, 29), revision of the stratigraphy of this area of the Sterkfontein deposits suggests that this specimen derives from the M5E infill (37) and thus should be attributed to either early *Homo* or *P. robustus*. Unfortunately, this specimen does not preserve enough of the proximal epiphysis to be taxonomically diagnostic and thus its attribution remains uncertain. Finally, although StW 311 is larger in absolute size than StW 522 (*SI Appendix*, Fig. S2*A*), both specimens show almost identical external morphology that has been previously interpreted as indicative of habitual bipedal locomotion (3, 29).

**Fig. 1.**
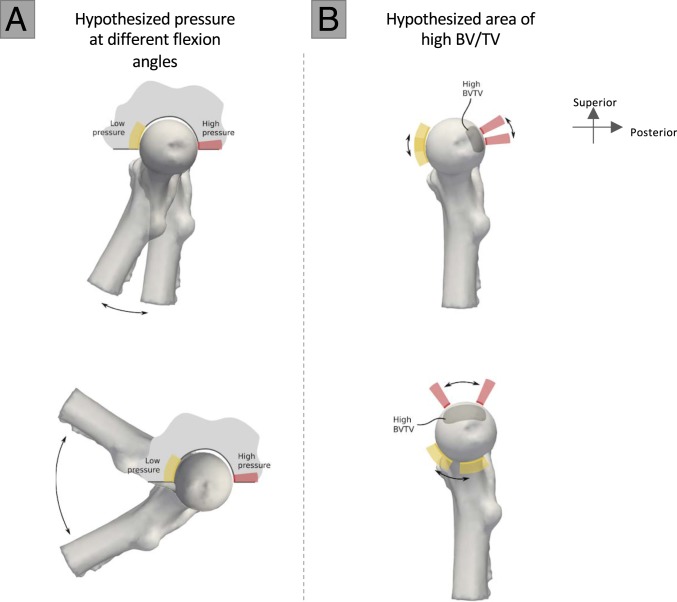
A schematic of hypothesized femoral head pressure and trabecular bone distribution at various flexion angles. (*A*) Hypothesized areas of high (pink) and low (yellow) pressure on the femoral head, based on how the femoral head fits within the incongruent hip joint at low flexion (e.g., bipedalism: *Above*) and moderate to high flexion (e.g., during terrestrial quadrupedalism and vertical climbing; *Below*). (*B*) The predicted resulting areas of high bone volume fraction (BV/TV). For a more detailed explanation refer to *SI Appendix*, Fig. S4.

To investigate the potential locomotor signals within the trabecular structure of the Sterkfontein hominin femoral specimens, we combine geometric morphometrics with trabecular analysis of the whole epiphysis (*SI Appendix*, Fig. S3*A*) to quantify and compare bone volume fraction at homologous locations across extant and fossil taxa. Based on predictions from joint morphology, hindlimb postures, and peak pressure data (39–43), we first investigate locomotor signals preserved in the trabecular structure of the femoral head of extant nonhuman great apes, including terrestrially knuckle-walking and arboreally climbing African apes (*Pan troglodytes verus n* = 11, *Pan t. troglodytes n* = 5, *Gorilla gorilla gorilla n* = 11), and orthograde arboreal orangutans (*Pongo* sp. *n* = 5). We predict that great apes will show a trabecular distribution (i.e., concentrations of high BV/TV) that is consistent with loading of the femoral head in both extended and highly flexed hip postures (Fig. 1), which occur during quadrupedalism, bipedalism, and vertical climbing. Second, we investigate the trabecular pattern in recent *Homo sapiens* (*n* = 11) and the femoral head of a fossil *H. sapiens* individual (Ohalo II H2). In contrast to great apes, we predict that recent and fossil *H. sapiens* will show a trabecular distribution that is consistent with posterior loading of the femoral head due to hip-joint incongruency and the use of habitual, more extended hip postures during bipedalism (Fig. 1). Finally, we assess the trabecular bone distribution in the femoral heads of StW 311 and StW 522, to determine whether they show functional signals in the femur consistent with ape-like, human-like, or distinct modes of locomotion. We predict that StW 522, attributed to *A. africanus* (36), will present a distinct trabecular pattern that shows similarities to both humans and great apes, given skeletal evidence suggesting that this taxon was a committed terrestrial biped that engaged in facultative arboreality (4, 7, 8, 13). Predictions for StW 311 are complicated by its taxonomic uncertainty and possible evidence for arboreality in *Paranthropus boisei* (44). If StW 311 represents *Homo*, then we predict a more human-like pattern; however, if it represents *Paranthropus* (and if one expects some level of arboreality in all members of this genus) then we predict that, like StW 522, it will show similarities to both humans and great apes.

## Locomotor Signals within the Proximal Femur of Nonhuman Great Apes

**Fig. 2.**
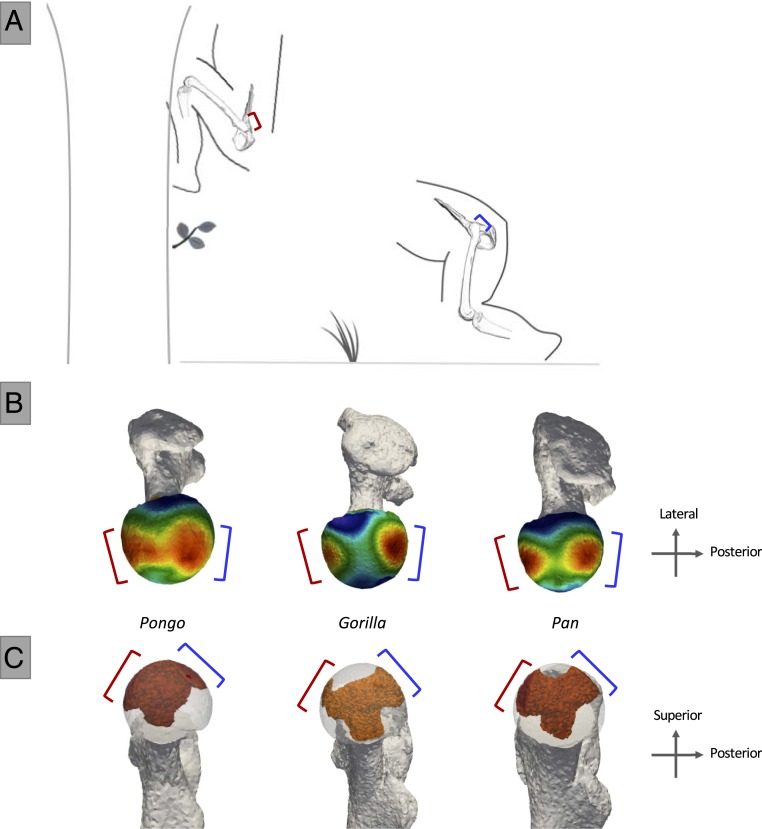
Nonhuman great ape hip flexion angles during terrestrial vertical climbing and quadrupedalism, and BV/TV distribution in the femoral head. (*A*) Great ape hip posture in maximum flexion (∼55° to 60°) during climbing (39), as well as joint posture at toe-off (∼110°) during terrestrial knuckle walking (40). Brackets indicate regions of presumed high pressure during large flexion (red, anterior) and slight flexion (blue, posterior). (*B*) Superior view of BV/TV distribution in the femoral head of *Pongo*, *Gorilla*, and *Pan*. High BV/TV is indicated in red and low BV/TV in blue. Note the two distinctly high BV/TV concentrations in *Gorilla* and the expansive distribution in *Pongo*, with *Pan* exhibiting an intermediate pattern. (*C*) Distribution of highest BV/TV values within the femoral head of *Pongo*, *Gorilla*, and *Pan*. Internal concentrations are visualized for BV/TV above the 80th percentile. This threshold was chosen to visualize the regions where the highest BV/TV is found within each specimen. Note that the internal high BV/TV forms an inverted cone in *Pongo*, and two convergent pillars in *Pan* and *Gorilla*.

Variation in the distribution of subchondral trabecular bone in the femoral head of nonhuman great apes is consistent with our predictions based on inferred joint position and pressure distribution in the hip during terrestrial as well as arboreal locomotion (Fig. 2 and *SI Appendix*, Fig. S4*C*; for average distribution maps for each taxon see *SI Appendix*, Fig. S3*B*; for trabecular architecture results see *SI Appendix*, Table S1; and for intertaxon comparisons of mean trabecular values see *SI Appendix*, Table S2). Extant nonhuman apes show two concentrations of high BV/TV across the surface of the femoral head (Fig. 2*B* and *SI Appendix*, Fig. S3*B*) that extend internally as two converging “pillars” or in the formation of an inverted cone (Fig. 2*C* and *SI Appendix*, Fig. S5). *Gorilla* has the most consistently well-separated regions of high BV/TV, followed by *Pan*, while *Pongo* has the least separated concentrations. The anterior concentration in all nonhuman apes is consistent with the presumed region of high pressure when hips are highly flexed during vertical climbing (39), while the posterior concentration is consistent with the region of high pressure when hips are more extended during terrestrial locomotion (40) (Figs. 1 and 2*A* and *SI Appendix*, Fig. S4*C*). Compared with *Gorilla*, there is a more expansive distribution in *Pan* and *Pongo* of high BV/TV across the superior aspect of the head, indicating a more variable pattern of joint positioning and pressure distribution. This is consistent with the use of more varied hip flexion angles during arboreal locomotion when needing to navigate complex forest canopies (41). The more restricted areas of BV/TV concentration in *Gorilla* suggest a more dichotomous joint positioning pattern, perhaps associated with reduced arboreality and/or large body size (39, 45).

## Locomotor Signals in Recent and Fossil *H. sapiens*

**Fig. 3.**
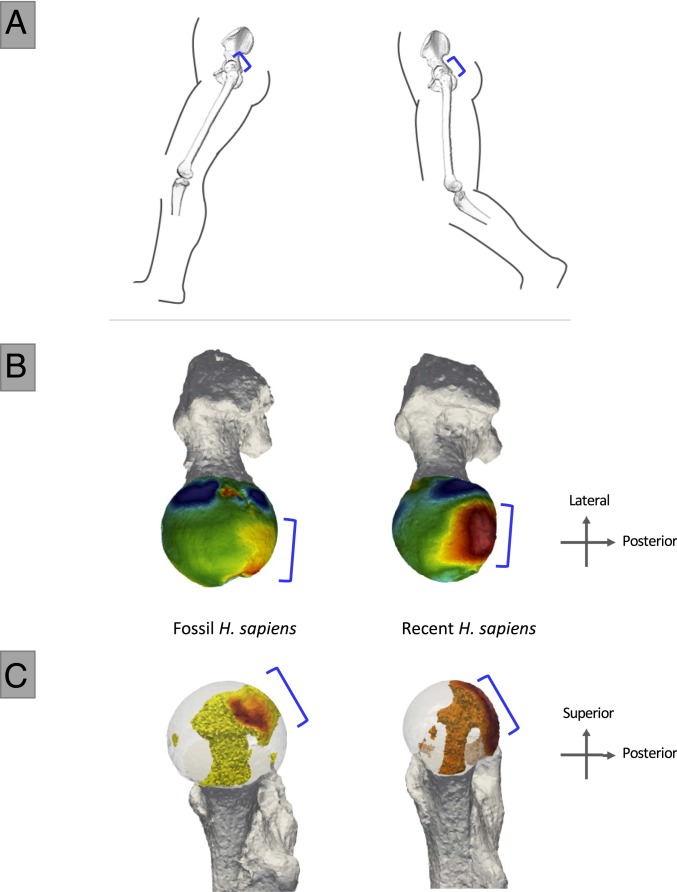
Human hip flexion angles during bipedal locomotion and BV/TV distribution in the femoral head of *H. sapiens*. (*A*) Modern human hip posture during bipedal walking at heel-strike (∼160°) and toe-off (∼175°), when ground reaction force is highest. Blue brackets indicate regions of inferred high pressure during bipedal walking. (*B*) Superior view of BV/TV distribution in the femoral head in fossil and recent *H. sapiens* is consistent with this loading prediction. High BV/TV is indicated in red and low BV/TV in blue. (*C*) Distribution of highest BV/TV values within the femoral head of *H. sapiens*. Internal concentrations are visualized for BV/TV above the 80th percentile. This threshold was chosen to visualize the regions where the highest BV/TV is found within each specimen. Note that the internal high BV/TV forms one pillar in *Homo*.

The pattern found in the femoral head of recent *H. sapiens* and Ohalo II H2 is distinct from that of other great apes, showing one region of high BV/TV located posteriorly and medially on the femoral head (Fig. 3 and *SI Appendix*, Fig. S3*B*). The region of high BV/TV corresponds to the region of highest pressure during a bipedal gait (Fig. 1 and *SI Appendix*, Fig. S4*B*) (42). Additionally, the extended range of intermediate values across the head is consistent with hip loading from positions of moderate flexion toward moderate extension (46). Intermediate BV/TV values continue along the inferior aspect of the femoral head (*SI Appendix*, Fig. S4*B*). Internally, *H. sapiens* shows the distinct feature of a single pillar of high BV/TV extending beneath the posterior-superior concentration toward the femoral neck (Fig. 3*C* and *SI Appendix*, Fig. S5).

## Trabecular Distribution Patterns and Locomotor Reconstruction of Sterkfontein Hominins

**Fig. 4.**
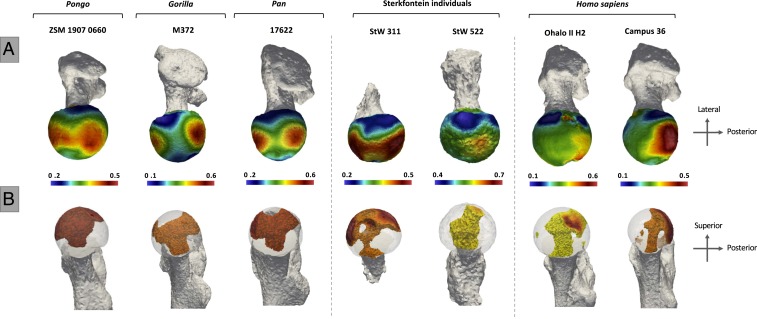
BV/TV distribution in the subchondral layer of the femoral head (*A*) and within the femoral head (*B*) in the extant and fossil taxa. StW 311 resembles the nonhuman ape-like patterns, while StW 522 resembles the human pattern (in addition to the Paleolithic specimen, Ohalo II, an example from our 19th century German cemetery sample [Campus 36] is used to represent the modern human pattern). Internal concentrations are visualized for BV/TV above the 80th percentile. This threshold was chosen to visualize the regions where the highest BV/TV is found within each specimen. Specimens are scaled to their own data range.

The two proximal femur fossil specimens from Sterkfontein show different trabecular patterns. The femoral head of StW 522 (attributed to *A. africanus*) exhibits one high BV/TV concentration along the superior aspect of the femoral head, located medially and close to the fovea capitis, that extends internally as a single pillar (Fig. 4 and *SI Appendix*, Figs. S3*C* and S5). This pattern, as well as the intermediate BV/TV values that continue across the inferior aspect of the femoral head, resembles that of *H. sapiens*. Despite the high BV/TV concentration being located slightly more anteriorly and mean femoral head trabecular parameters (e.g., DA, trabecular number, and thickness) falling within the extant ape range (*SI Appendix*, Fig. S2*B*), the BV/TV distribution pattern of this specimen is almost identical to *H. sapiens*. Contrary to single trabecular parameters (33), BV/TV distribution patterns in the femur (30) and other bones (28, 34), have been shown to distinguish between great apes with different locomotor repertoires; therefore, these results suggest that StW 522 used a similar bipedal gait to *H. sapiens*.

In contrast to StW 522, the geologically younger proximal femur StW 311 shows a more ape-like trabecular pattern. This individual has two concentrations of high BV/TV along the superior aspect of the femoral head that extend internally toward the neck (Fig. 4). The ape-like anterior concentration suggests that, in addition to typical bipedalism, this individual frequently adopted a highly flexed hip posture. Furthermore, in contrast to previous findings (29), mean femoral head trabecular parameters of StW 311 fall consistently within the *Pan* range (*SI Appendix*, Fig. S2*B*). Although these mean values may obscure or homogenize the variation in each trabecular parameter within the femoral head, our results show that StW 311 has low anisotropy and high BV/TV compared to the typical pattern in sedentary *H. sapiens* (29).

**Fig. 5.**
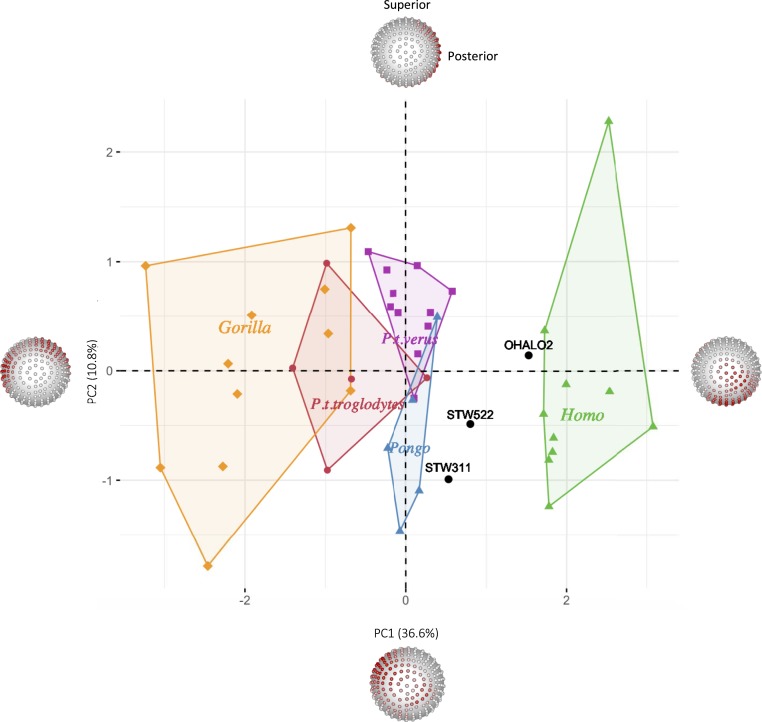
PCA of the relative BV/TV distribution in the femoral head. PC1 (*x* axis) explains 36.6% of the variation, while PC2 (*y* axis) explains 10.8% of the variation. Landmarked spheres depict RBV/TV regions of highest loading (red) on each PC axis. RBV/TV values on the inferior aspect of the head have the highest positive loading on PC1, (separating *H. sapiens* from the nonhuman apes) and RBV/TV values on two regions across the superior aspect of the head have the highest negative loading (being most clearly expressed in *Gorilla*). PC2 does not separate taxa but is driven by high RBV/TV posteriorly versus anterosuperiorly. Considerable variation, specifically in *Gorilla*, could relate to sexual dimorphism and differences in habitual hip angles between the sexes.

To further assess the trabecular architecture of the Sterkfontein femoral specimens compared to extant apes and recent and fossil *H. sapiens*, we conducted an analysis of relative BV/TV (RBV/TV) distribution in the femoral head using geometric morphometric techniques. Two hundred and forty-two landmarks and semilandmarks were defined on the subchondral femoral head. Subsequently, BV/TV values were extracted at each subchondral landmark and were standardized by the mean BV/TV value of all subchondral landmarks extracted from that specimen, resulting in a relative bone volume fraction (RBV/TV). RBV/TV values were then statistically compared between taxa, to identify relative differences in their distributions, rather than raw differences of trabecular volume values. Fig. 5 presents a principal component analysis (PCA) of the landmark-based RBV/TV distribution across the surface of the femoral head of all taxa. Consistent with the overall patterns described above for the extant taxa, along PC1 *Gorilla* is distinguished from *Pan* and *Pongo* species, which cluster together, while *H. sapiens* is clearly separated from all other apes. Permutational MANOVA tests of the first three principal components reveal that the distributions of all taxa differ significantly, except that of *Pan t. troglodytes* from the other nonhuman apes (*SI Appendix*, Table S3). Ohalo II H2 falls just outside the recent human distribution but shows the same subchondral trabecular pattern. This is consistent with the fact that our *H. sapiens* sample does not include sufficient variation in terms of geographic distribution and behavioral diversity. Both StW 522 and StW 311 fall out as intermediate between *H. sapiens* and *Pan*/*Pongo*, but StW 311 is closer to the nonhuman apes. This result reflects the more concentrated subchondral BV/TV distribution in STW 522 compared to the more dispersed anterosuperior to posterosuperior concentration in STW 311 (*SI Appendix*, Fig. S3*C*). It also further supports the inferred differences in loading between these two specimens as evidenced by their internal BV/TV distribution reported above.

## Discussion

In this study, we demonstrate that known differences in the locomotor behavior of nonhuman apes and humans are reflected in the trabecular structure of the femur. We also provide substantive evidence that early Pleistocene fossil hominins from Sterkfontein, who existed at different times, were using distinct forms of locomotor behavior. Contrary to our predictions, *A. africanus* StW 522 showed a distinctly human-like trabecular bone distribution. This result suggests that potential bouts of climbing/arboreality were not practiced at a frequency similar to that responsible for the distinctive pattern present in nonhuman great apes. Our findings could be considered support for interpretations that other *Australopithecus* species (i.e., *A. afarensis*) were committed terrestrial bipeds (9, 13); however, they should also be evaluated within the context of evidence from the upper limb in *A. sediba* that is interpreted to represent frequent bouts of arboreality (47). If the trabecular bone is adequately imageable, analyses of the near complete skeleton of *Australopithecus* sp. StW 573 (13) (Little Foot) may further elucidate the locomotor behavior of Sterkfontein hominins.

Given the similar external morphology between the Sterkfontein proximal femora in our sample, trabecular evidence that StW 311 frequently used highly flexed hip postures typical of climbing may be unexpected, but follows our prediction for this specimen based on its potential taxonomic affinity and associated postcranial evidence for some degree of arboreality in either *Paranthropus* or early *Homo*. This result is also consistent with paleoenvironmental reconstructions from faunal evidence (48) that suggest that there was significant tree coverage near the Sterkfontein Caves during the accumulation of the Member 5 East infill, but drier climate than Member 4 (*SI Appendix*). However, as is common in vertebrate paleontology, it is difficult to place individuals in a particular part of a diverse landscape. There are various ways in which StW 311 may have come to be preserved at Sterkfontein, including carnivore accumulation, water transport, or death traps (48). Thus, although a climbing signal is most often associated with arboreality in a wooded environment, climbing within a karstic environment is also a possibility. Additionally, it is uncertain if other highly flexed-hip behaviors, such as frequent squatting, could result in a similar trabecular distribution pattern to that of climbing in apes. This could be explored in human samples with evidence for squatting in the lower limb bones (e.g., squatting facets, ref. 49). However, our expectation is that positional loading during squatting is unlikely to result in comparisons between human groups resembling the dichotomous pattern we find between humans and nonhuman apes.

Our results from the trabecular analysis of StW 311 add to those previously described in a distal tibia specimen (StW 567) from the Member 5 East infill. Barak et al. (27) found that this individual had human-like trabecular orientation, that differs from chimpanzees, reflecting the use of less dorsiflexed ankles. However, the mean trabecular parameters of this specimen were not distinctly human-like. For example, BV/TV in the two studied volumes of interest of StW 567 was higher than both *H. sapiens* and *P. troglodytes*; DA was more similar to *P. troglodytes*; and trabecular number, separation, and connectivity were intermediate between the two extant taxa. The lack of certainty on the taxonomic affinity of StW 567 introduces difficulties in the interpretation of these results, as we do not know if it belongs to the same taxon as StW 311 and the Member 5 East infill contains both *P. robustus* and early *Homo* fossils. An associated lower limb that included both the femur and tibia may elucidate the likelihood that these two specimens could sample the same taxon.

Based on our predictions, evidence for the frequent use of a highly flexed hip joint in the StW 311 individual could be evidence in support of this specimen belonging to *Paranthropus*, rather than *Homo*. However, there are a number of important points that must be considered. First, evidence for arboreality in *P. boisei* is limited to a scapula, which shows both arboreal and nonarboreal features (44), a distal humerus (50), and a proximal radius (51). Additionally, given the lack of overlapping postcranial evidence from relevant regions of the appendicular skeleton, there is no unequivocal evidence for a shared locomotor repertoire between eastern and southern African *Paranthropus* species. Second, postcranial signals of arboreality have been noted in some early *Homo* specimens, such as Olduvai Hominid (OH) 62 (19), and it is thus conceivable for StW 311 to represent *Homo* and show evidence for arboreality. Finally, two proximal femora, Swarktrans (SK) 3121 and Swartkrans Wits (SKW) 19, which could also be either *P. robustus* or early *Homo*, were not included in the main study due to our inability to segment their trabecular structure with sufficient confidence due to taphonomic alteration (*SI Appendix*, Fig. S6). However, there is potential evidence from the internal BV/TV distribution (*SI Appendix*, Fig. S6*C*) for a human-like, single concentration in these specimens. Additional scanning of these specimens could allow a reassessment of this potential patterning. Determining the taxonomic affiliation of not only StW 311, but also SK 3121 and SKW 19 remains crucial as it will have clear implications, and perhaps explanations, for niche differentiation between *Homo* and *Paranthropus* who differ in gnathic morphology, but less so in dental microwear and dietary isotopic data (52).

Finally, the results of this study add to the increasing evidence for locomotor diversity in the Plio-Pleistocene hominin record including a mix of primitive and derived features in Little Foot (13), *A. sediba* (24), and *H. naledi* (26), the abducted hallux in the Burtele foot (53), and more ape-like than hominin-like lower limb morphology in *Ardipithecus ramidus* (5). We suggest that future studies of internal bone structure (both cortical distribution and trabecular architecture) will be crucial to clarifying the diversity of locomotor behaviors that characterized various hominin lineages.

## Materials and Methods

### Sample, Segmentation, and Trabecular Architecture Analysis.

In this study we used microcomputed tomographic scans to analyze trabecular architecture in the femoral head of five extant ape taxa (*P. troglodytes verus n* = 11, *P. t. troglodytes n* = 5, *Pongo* sp. *n* = 5, *G. gorilla n* = 11, and *H. sapiens n* = 10) and three fossil specimens (StW 311, StW 522, and Ohalo II H2), detailed in *SI Appendix*, Table S4. Samples were provided by the Powell-Cotton Museum (*Gorilla*), the Max Planck Institute for Evolutionary Anthropology and the Smithsonian Museum of Natural History (*Pan*), the Mammal collection of the Zoologische Staatssammlung München (*Pongo*), and Georg-August-Universitaet, Goettingen, Germany (*H. sapiens*). Because these are historical museum collections, informed consent and institutional review board approval were not required. The *P. troglodytes verus* individuals came from the Taï forest, while four of the *P. t. troglodytes* individuals came from Gabon and one from Cameroon. We included two subspecies of *Pan* to show the sensitivity of our method in detecting differences in BV/TV distribution between closely related taxa with few behavioral differences. All *Gorilla* individuals were western lowland gorillas, and 13 came from Cameroon while 1 came from the Democratic Republic of the Congo. The *Pongo* sample consisted of one *Pongo abelii* individual, three *Pongo pygmaeus* individuals, and one unspecified. All nonhuman apes were wildshot. The *H. sapiens* individuals came from two 19th to 20th century cemeteries in Germany. Several South African hominin specimens (e.g., SK 3121, SKW 19, SK 82, and SK 97) were excluded from our analysis because of difficulties in obtaining an accurate representation of the trabecular structure or limited preservation that excluded homologous landmarking (*SI Appendix*, Figs. S6 and S7). All individuals were adult and showed no signs of pathologies. Prior to analysis, all specimens were reoriented to approximate anatomical positions, as well as cropped and resampled when necessary using AVIZO 6.3 (Visualization Sciences Group, SAS).

Segmentation of bone from air was performed using the Ray Casting Algorithm (54) for the extant sample and the medical image analysis-clustering algorithm (55) for the fossil sample (*SI Appendix*, Fig. S1). The latter was used for fossils as it allows more accurate separation of trabecular bone from surrounding inclusions. Trabecular architecture was analyzed in medtool 4.1 (56), following previously described protocol (57). Three-dimensional tetrahedral meshes with a 1-mm mesh size were created using CGAL 4.4 (Computational Geometry; ref. 58) and BV/TV values, which were obtained using a sampling sphere with a 7.5-mm diameter, on a 3.5-mm background grid, were interpolated onto the elements creating BV/TV distribution maps. Internal BV/TV distribution was visualized in Paraview (59) above selected percentiles which were calculated for each femoral head using the quantile function in R v3.4.1 (60). The visualization shows where the highest 15%, 20%, and 25% of the BV/TV values lie within that femoral head (*SI Appendix*, Fig. S5). This method was chosen to ensure that the selected thresholds were not affected by outliers and that isolated patterns were comparable between specimens.

The subchondral surface of the resulting 3D models was extracted and smoothed using Screened Poisson surface reconstruction in MeshLab (61) in preparation for landmarking.

### Landmarking and BV/TV Values Extraction.

Initially, fixed landmarks were selected for the femoral head. Intraobserver error for the fixed landmarks was tested by placing the landmarks on three specimens of the same taxon on 10 nonconsecutive occasions. Five fixed landmarks were identified on the femoral head; one point in each direction of the head-neck border (most anterior, most posterior, most lateral, and most medial) at the midpoint and one on the surface of the femoral head, at the center of the four corner landmarks (*SI Appendix*, Fig. S3*A*). Four semicurves were defined between the fixed landmarks along the femoral head-neck boundary, each containing seven landmarks. Subsequently, 208 semilandmarks (62) were defined on the surface of the femoral head. These were evenly spaced landmarks extending across the whole femoral articular surface. Thirty-two of the semilandmarks were placed between the fixed landmarks on the head-neck boundary (1–4) and the fifth landmark at the midpoint of the corner landmarks, thus dividing the femoral head into quarters. The remaining landmarks were placed covering the surface of the quarters. Further description of the landmarks is given in *SI Appendix*, Table S5.

The fixed and curve landmarks were manually defined on all specimens, while the surface semilandmarks were defined on one specimen and then projected on all other specimens using the Morpho package (63) in R v3.4.1 (60). After manual inspection of the projected landmarks on each specimen, the landmarks were relaxed on the surface minimizing bending energy. Subsequently, the Morpho package was used to slide the surface and curve landmarks minimizing Procrustes distance. A medtool 4.1 custom script was used to interpolate BV/TV values to landmark coordinates from the closest neighboring tetrahedron in distribution maps of each specimen. RBV/TV values were calculated for each landmark by dividing landmark BV/TV values by the average of all BV/TV landmark values of each individual. Relative values were used for the statistical analysis to ensure intertaxon comparisons focused on differences in the distribution rather than magnitude.

### Statistical Analysis.

Statistical analysis was performed in R v3.4.1 (60). A PCA was used to visualize interspecific differences in RBV/TV distributions. To exemplify the sensitivity of this method we evaluated the distributions of the *Pan* subspecies separately. Bonferroni-corrected, one-way pairwise permutational MANOVA tests of the first three principal components were used to test whether observed differences between the taxa in the PCA are significant (*P* ≤ 0.05). The three first components were chosen as they explained high percentages of the variation and together amounted to more than ∼50%.

### Data and Materials Availability.

The data and materials can be accessed at https://data.kent.ac.uk/id/eprint/109.

## Supplementary Material

Supplementary File
