# Supplementary material for: Evidence for habitual climbing in a Pleistocene hominin in South Africa
Source: Proc Natl Acad Sci U S A. 2020 Mar 30;117(15):8416–23. doi: 10.1073/pnas.1914481117 (PMC7165455; doi:10.1073/pnas.1914481117)
Supplement: Supplementary File [file pnas.1914481117.sapp.pdf]

Supporting text

StW 522 (P45 26'1''-27'1'' below datum)[1] derives from Member 4 (the bulk of which is exposed east of grid line 49) that has been dated broadly to 2.8 to 2.0 Ma[2] and has been attributed to *A. africanus*[3]. Paleoenvironmental reconstructions suggest that over the time of formation of Member 4, habitats included closed forest and more open grassland in the proximity of the cave opening[4-10].

The StW 311 proximal femur derives from the stratigraphically complex eastern end of Member 5 (named Member 5 East – M5E)[11] at Sterkfontein (R52; 14'5''-15'5'' below datum). In the north of M5E, where StW 311 was discovered, two infills are recognised[12], both of which are artefact- and hominin-bearing. In the lower infill unit of M5E, the *P. robustus*- and Oldowan artefact-bearing deposit has recently been dated to 2.18 Ma[13] but has previously been suggested to date from 1.7-1.4 Ma[11]and 1.4-1.2 Ma[2,14]. The deposit has been spatially constrained through the presence of artefacts to grid lines 49-58 and depths of 36'10'' below datum in the deepest square to 22' below datum at its shallowest[11]. Above 20' below datum, the M5E Early Acheulean-bearing deposit spans from grid line 49 west to about line 58, where the Acheulean and lower part of M6 have been eroded and replaced by the Middle Stone Age Post-Member 6 deposit[11]. West of grid line 64, the Acheulean-bearing deposits continue as M5W Acheulean. Bifaces and early Acheulean stone tools are associated with StW 80, attributed to *Homo ergaster*[15]. This unit has been dated to 1.7-1.4 Ma based on stone tool typology and the presence of *H. ergaster*[11], although Herries and Shaw[2] have suggested a younger date of 1.3-1.1 Ma. In the upper part of the M5E Acheulean, which is decalcified and solution cavities have mixed sediments and contaminated the deposit[11,16]. The StW 311 proximal femur has been attributed to *A. africanus* in several studies[17,18]. However,

based on Kuman and Clarke's revision[11] of Sterkfontein's stratigraphy in this area, StW 311 derives from the Member 5 East infill and thus is more likely to be associated with Member 5 (than Member 4) and therefore attributed to *P. robustus* or early *Homo* (including *H. ergaster*). Both Member 5 infills (M5E Oldowan and M5 Acheulean; M5E and M5W) were formed during periods when open savannah environments dominated with periods and pockets of wooded grassland[5,11,19-24] and locally well-developed soils[25].

Supporting Figures

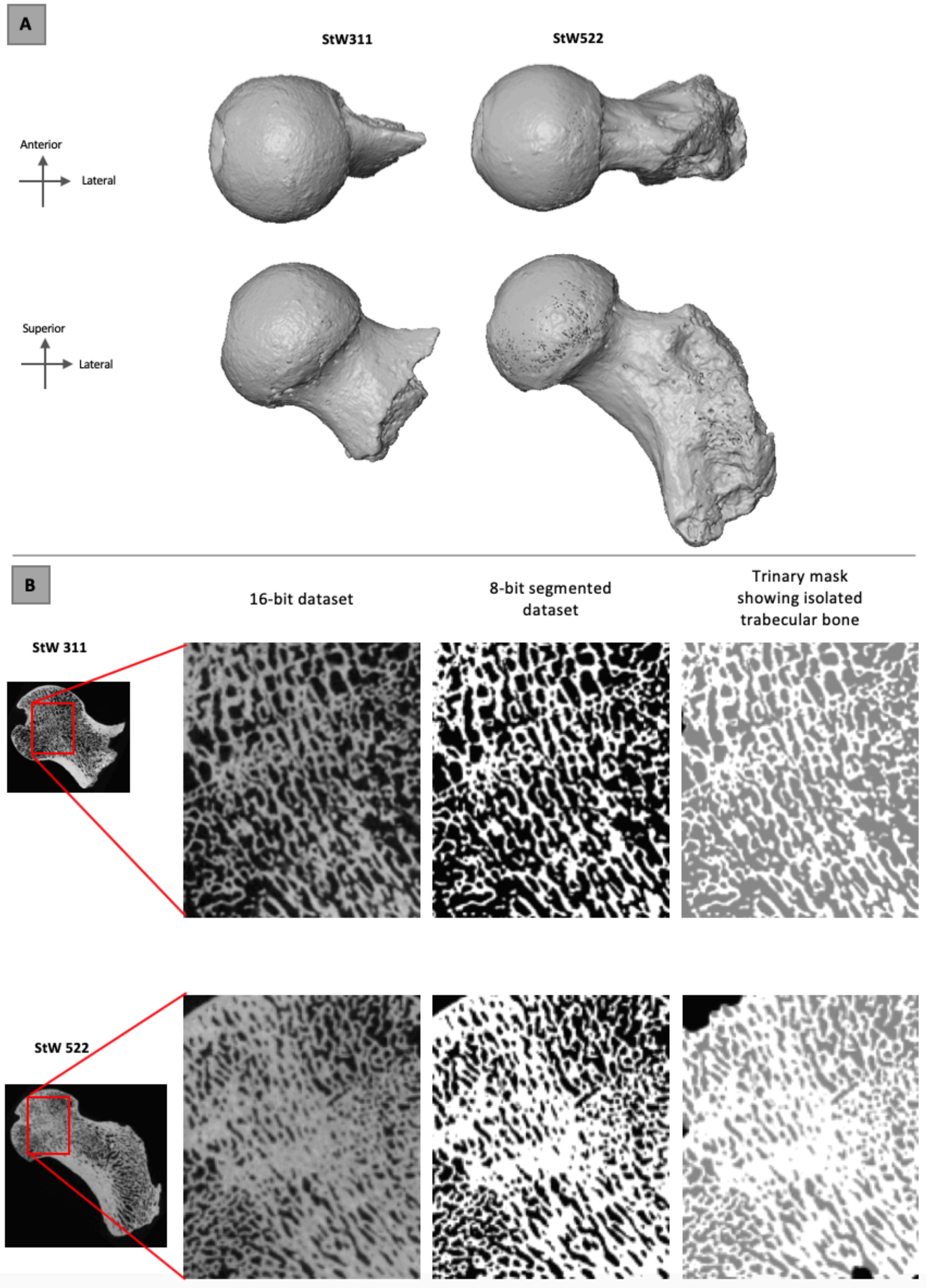

Supporting Figure 1. External (A) and internal (B) morphology of StW 311, and StW 522. (A) Three-dimensional models showing the superior (top) and posterior (bottom) views. (B) High resolution images of the preserved trabecular structure (left), segmented bone (middle) and trinary mask showing isolated trabecular structure (right).

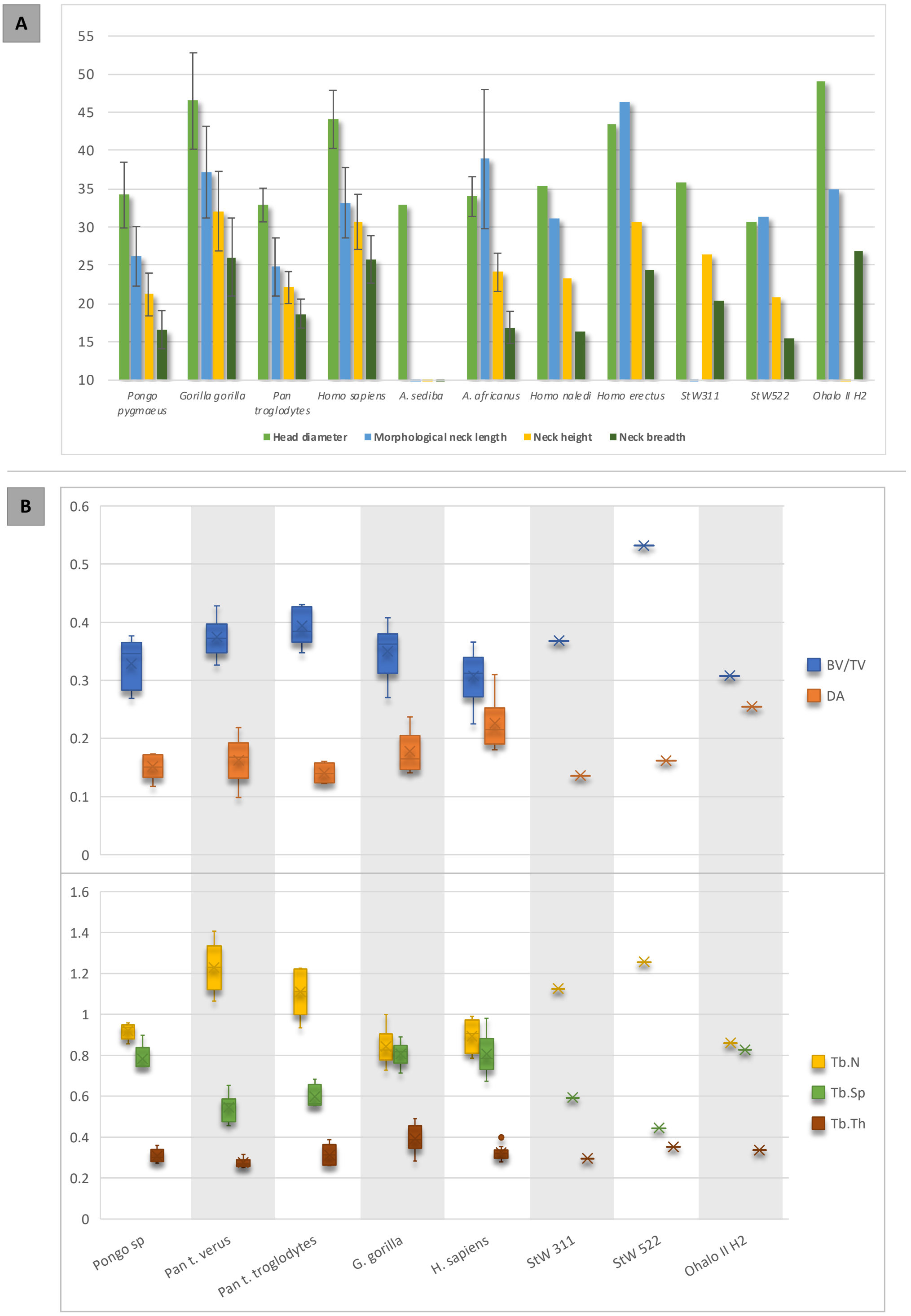

Supporting Figure 2. Comparative femoral measurements (mm) for extant and extinct taxa (A) and trabecular parameters (mm-Tb.Sp, Tb.Th, 1/mm-Tb.N) for the sample (B). (A) Columns represent mean values for each femoral measurement and error bars represent the standard deviation. Comparative femoral measurements were taken from Harmon[17], except for *A. sediba* which were taken from DeSilva et al.[26] Suppl. Online Material, *H. naledi* and *H. erectus* which were taken from Marchi et al.[27] and Ohalo II H2 which were taken from Hershkovitz et al. [28](B) Trabecular parameters quantified over the entire femoral head for the extant taxa and the fossils. The trabecular structure of StW 522 has likely been influenced by taphonomic processes and although we are confident in the pattern of distribution of BV/TV the absolute values of some parameters may have been slightly affected.

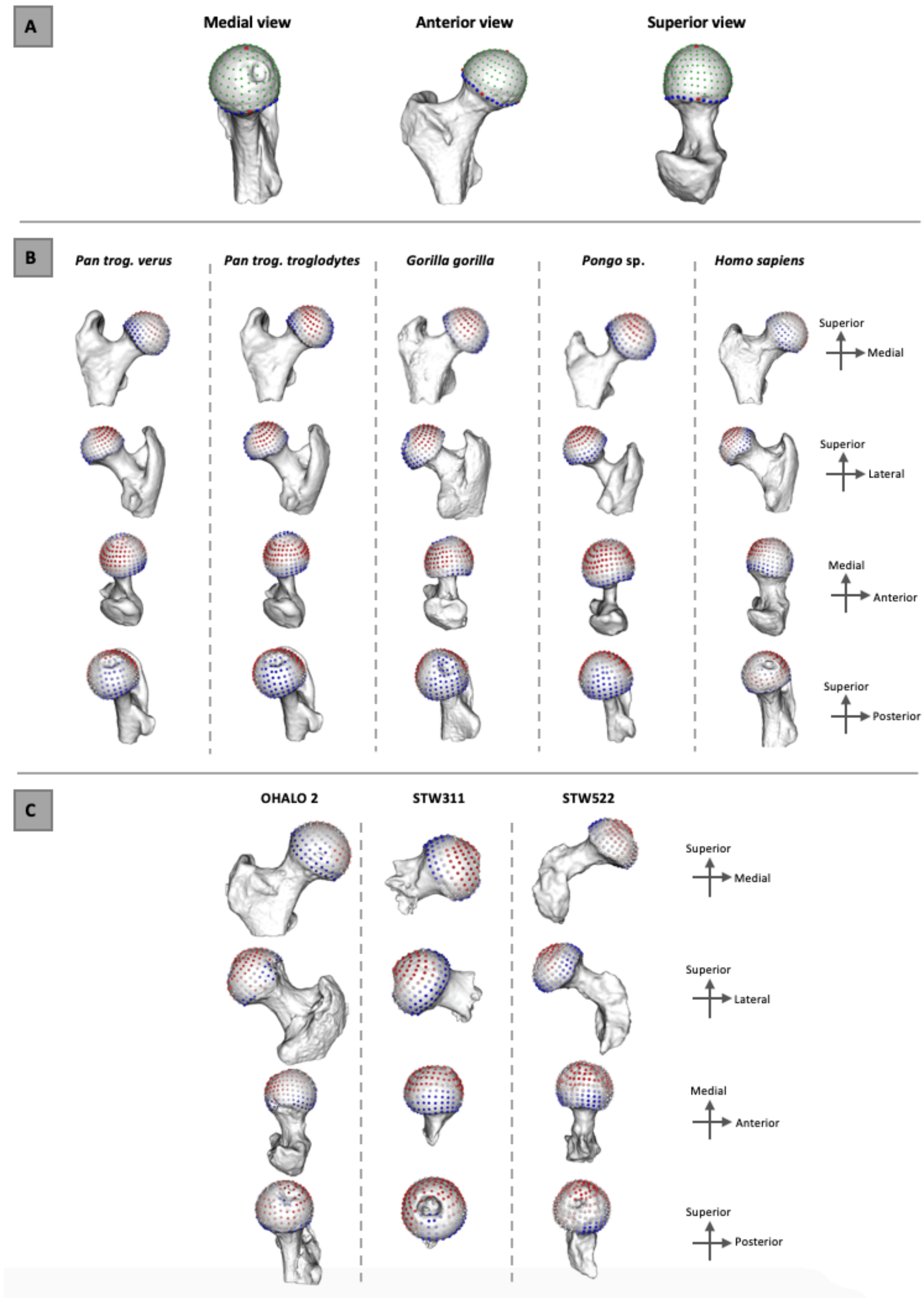

Supporting Figure 3. Landmarking and results for the femoral head. (A) Landmarks used for the analysis of the femoral head trabecular structure. Fixed landmarks are indicated in red, semi-landmarks on curves are indicated in blue and surface semilandmarks are indicated in green. (B) Taxon-average RBV/TV distributions over the femoral head in the extant taxa. High RBV/TV is indicated in red and low RBV/TV in blue. (C) RBV/TV distributions over the femoral head in the fossils.

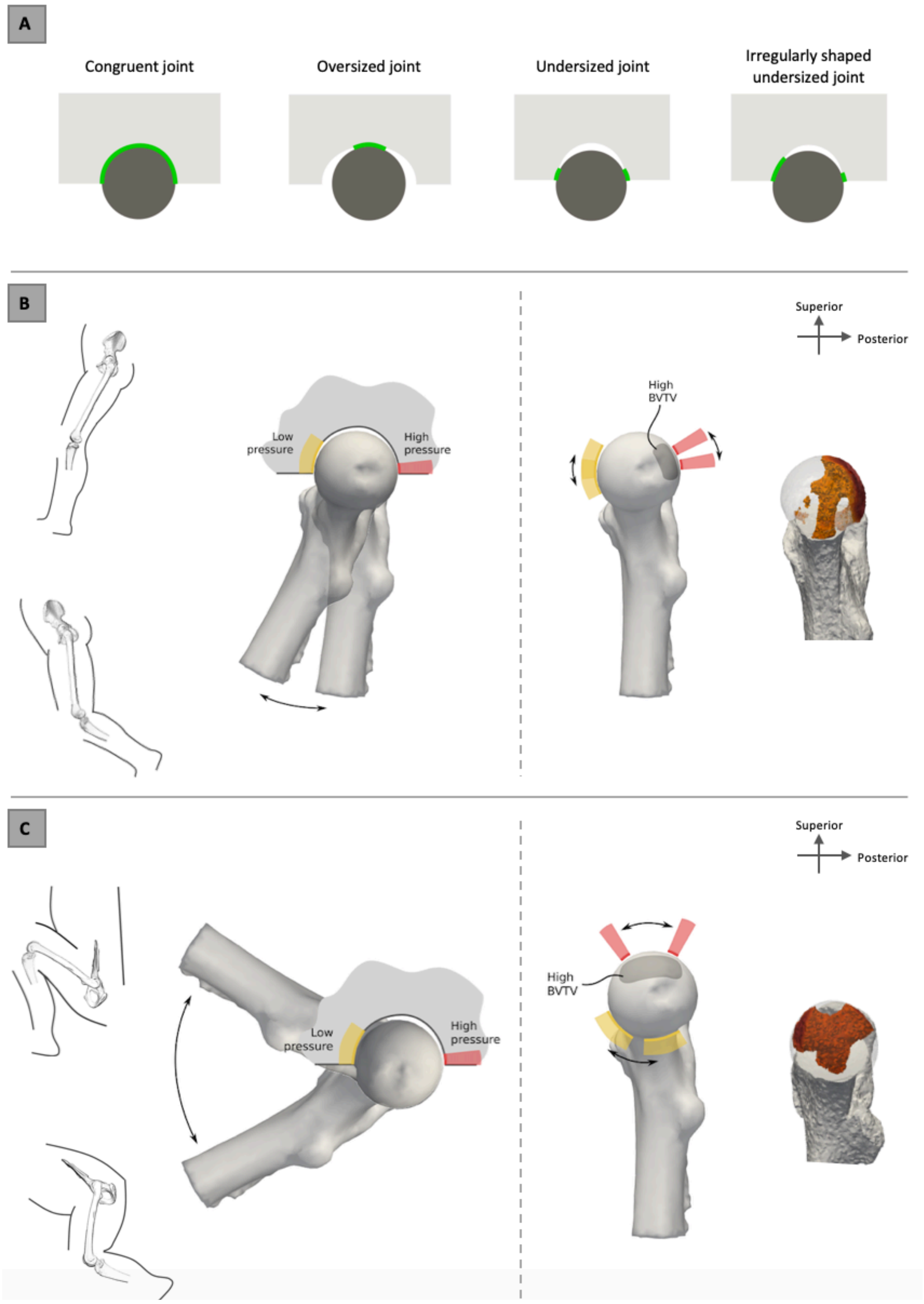

Supporting Figure 4. Contact surface in congruent and incongruent joints (A), as well as presumed hip pressure distribution in humans (B) and non-human apes (C) during habitual activities. (A) Contact area (shown in green) between the femoral head and the acetabulum in different joint types. In humans, the hip is an incongruent, undersized joint with an irregularly shaped lunate surface[29,30]. We hypothesise that this results in differently sized contact areas in the anterior and posterior regions of the femoral head. In this study this is assumed to be similar across ape taxa. (B) Human hip joint angles at touchdown and toe-off (left) and predicted pressure distribution on the femoral head (right), consistent with *in vivo* data[31]. The human BV/TV distribution map (far right) matches the predicted pattern based on joint morphology and pressure distribution. (C) Non-human ape hip joint angles during climbing and toe-off (left) and predicted pressure distribution on the femoral head (right). The non-human ape BV/TV distribution map matches the predicted pattern.

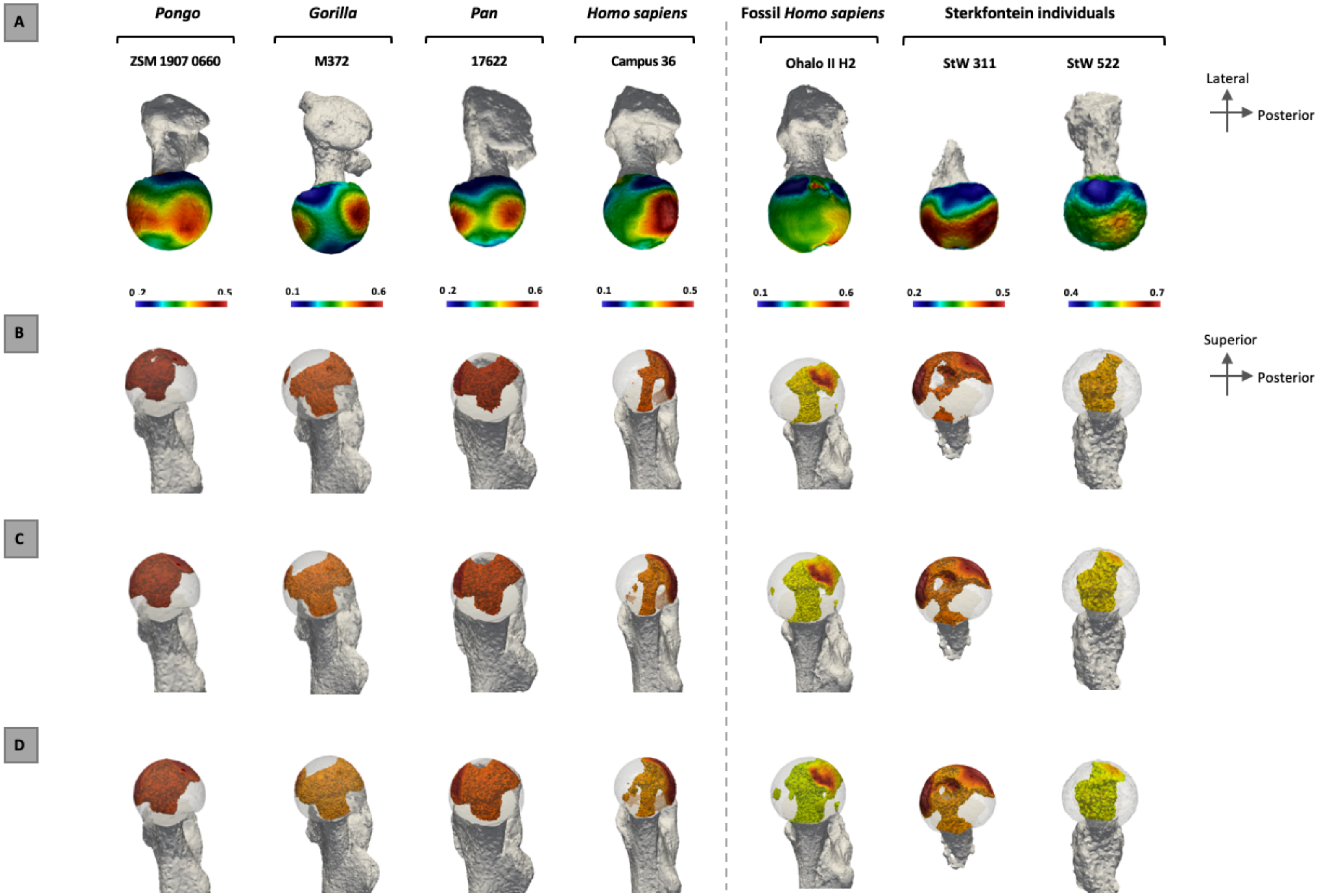

Supporting Figure 5. BV/TV distribution in the subchondral layer of the femoral head (A) and within the femoral head (B-D) in the extant and fossil taxa. Internal high BV/TV is shown above the 85th (B), 80th (C) and 75th (D) percentile in each individual. Specimens are scaled to their own range.

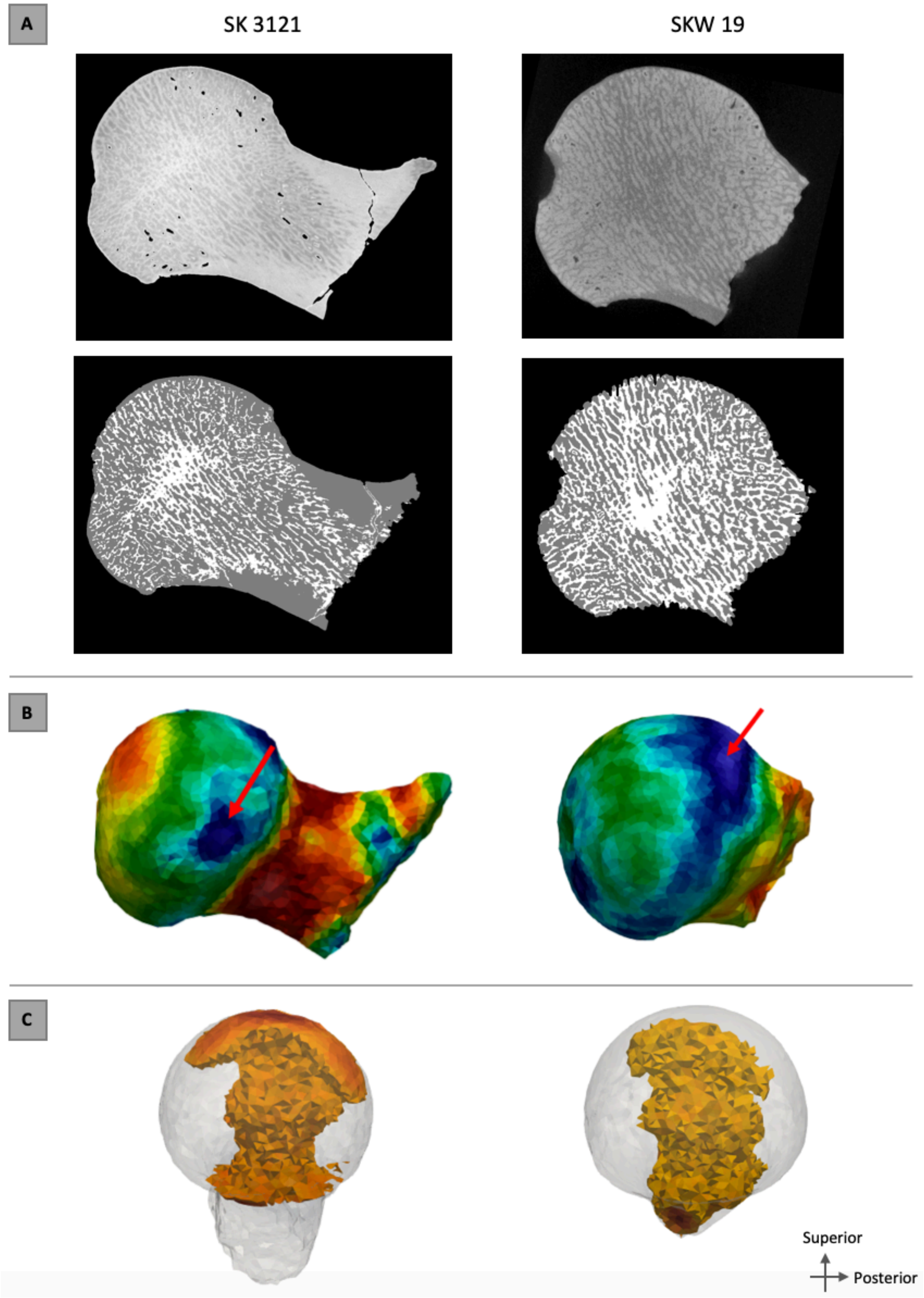

Supporting Figure 6. South African hominin specimens which were not used in the analysis but were processed. (A) Original scan and trinary mask of SK 3121 and SKW 19. When segmented, taphonomic alterations to trabecular structure became clear and therefore, these fossils could not be used in our analysis. Furthermore, SKW 19 does not preserve enough of the femoral neck for accurate landmarking. (B) BV/TV distribution maps showing problematic regions (red arrows). (C) BV/TV distribution within the femoral head above the 80th percentile (potentially shows single pillar in these specimens).

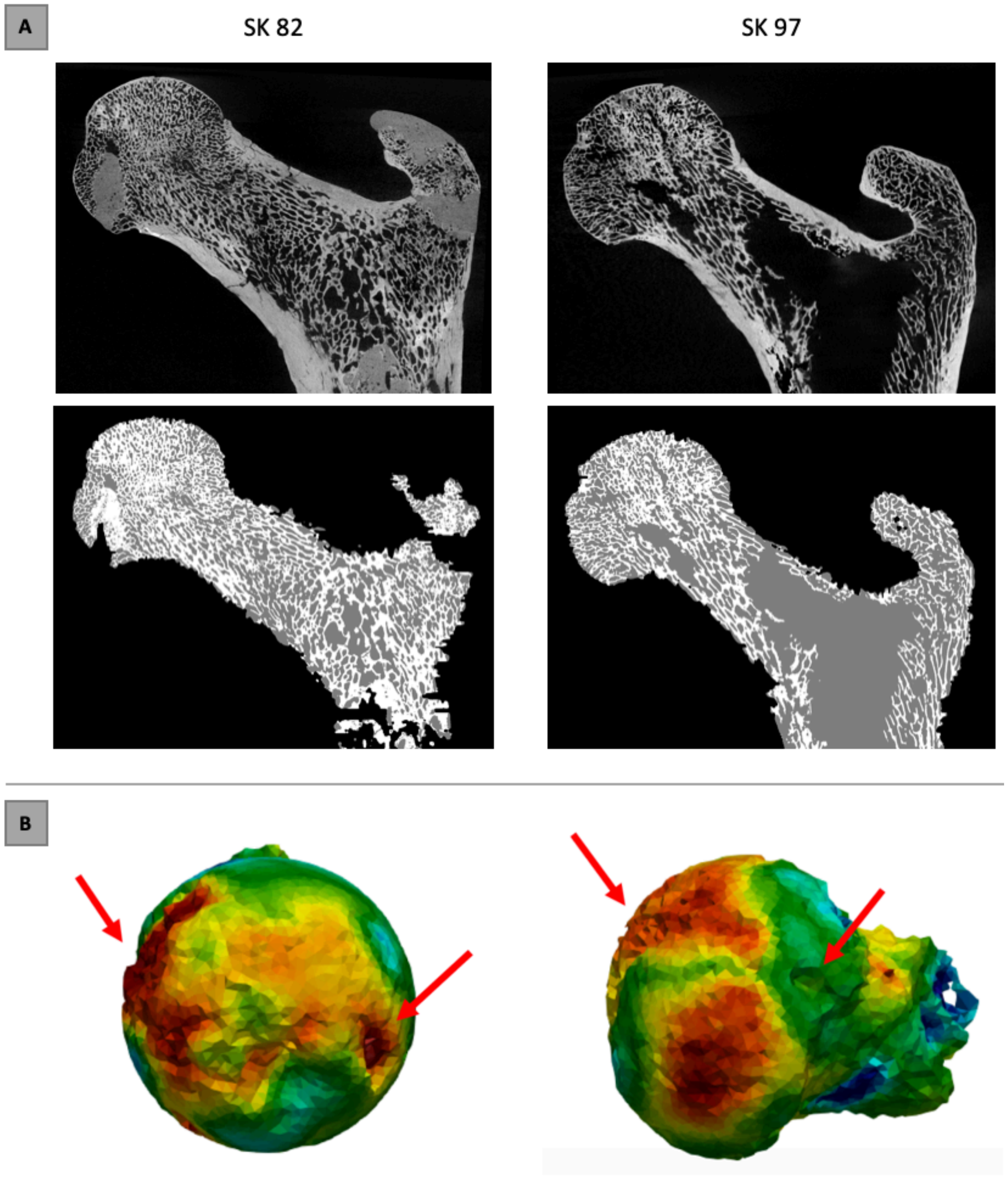

Supporting Figure 7. Additional South African hominin specimens which were not used in the analysis but were processed. (A) Original scan and trinary mask of SK 82 and SK 97. These specimens did not preserve enough of the trabecular structure for meaningful comparisons with the extant sample. (B) BV/TV distribution maps shown problematic areas (red arrows). These indicate that after segmentation the trabecular structure presents gaps and cannot be evaluated accurately.

Supporting Tables

Supporting Table 1. Trabecular architecture results. Mean, standard deviation (in parentheses) and coefficient of variation for five trabecular parameters quantified throughout the femoral head.

Taxon	*P.t. verus*	CV	*P.t. trogl.*	CV	*Gorilla*	CV	*Pongo*	CV	*Homo*	CV
BV/TV	0.37 (0.03)	7.96	0.39 (0.03)	8.52	0.35 (0.05)	12.91	0.33 (0.04)	13.38	0.30 (0.04)	13.98
DA	0.16 (0.04)	24.04	0.14 (0.02)	12.36	0.18 (0.03)	18.85	0.15 (0.02)	14.66	0.23 (0.04)	18.28
Tb.N (1/mm)	1.23 (0.11)	9.26	1.11 (0.12)	10.89	0.84 (0.08)	9.52	0.92 (0.04)	4.36	0.89 (0.08)	9.22
Tb.Sp (mm)	0.54 (0.06)	11.17	0.60 (0.06)	9.51	0.81 (0.06)	7.47	0.78 (0.07)	8.43	0.80 (0.10)	12.07
Tb.Th (mm)	0.28 (0.02)	8.42	0.31 (0.05)	16.91	0.39 (0.07)	17.11	0.31 (0.03)	10.89	0.32 (0.04)	10.97

Supporting Table 2. Intertaxon pairwise comparisons of mean femoral head trabecular parameters. Bonferroni corrected p-values are given for each comparison.

	Pan t.v.-Pan t.t.	***Pan t.v.-Gorilla***	***Pan t.t.-Gorilla***	***Pan t.v. - Pongo***	***Pan t.t. - Pongo***	***Pan t.v. - Homo***	***Pan t.t. - Homo***	***Gorilla - Pongo***	***Gorilla - Homo***	***Pongo - Homo***
**BV/TV**	1	1	0.517	0.517	0.317	0.002	0.018	1	0.336	1
**DA**	1	1	0.380	1	1	0.024	0.005	1	0.104	0.005
**Tb.N**	0.687	2.8e-05	0.009	0.005	0.556	2.8e-05	0.055	0.517	1	1
**Tb.Sp**	1	2.8e-05	0.005	0.005	0.079	2.8e-05	0.009	1	1	1
**Tb.Th**	1	0.000	0.380	0.380	1	0.005	1	0.192	0.158	1

Supporting Table 3. Intertaxon pairwise permutational MANOVA tests of the first three principal components of the PCA. Bonferroni corrected p-values and Pseudo-*F* (in brackets) are given for each comparison in the femoral head.

Taxon	*Pongo sp.*	*G. gorilla*	*P.t. verus*	*P.t. troglodytes*	*H. sapiens*
Pongo sp.		0.012	0.025	1	0.002
G. gorilla	(7.9)		0.001	1	0.001
P.t. verus	(6.4)	(16.1)		0.208	0.001
P.t. troglodytes	(2.5)	(2.2)	(3.9)		0.003
H. sapiens	(7.2)	(34.6)	(17.3)	(12.2)	

Supporting Table 4. Study sample composition, sex and resampled voxel size range in both epiphyses.

**Taxon**	**N**	**Sex**	**Proximal voxel size (mm)**	**Collection or Site, Institution**
Pan troglodytes verus	11	7 female, 4 male	0.04-0.05	Taï Forest collection, Max Planck Institute for Evolutionary Anthropology, Leipzig, Germany.
Pan troglodytes troglodytes	5	3 female, 2 male	0.05	Smithsonian National Museum of Natural History in Washington, DC, USA.
Gorilla gorilla gorilla	11	6 female, 5 male	0.05-0.08	Powell-Cotton Museum, UK.
Pongo sp.	5	5 female	0.04-0.045	Zoologische Staatssammlung München, Germany.
H. sapiens	10	3 female, 6 male, 1 N/A	0.06-0.07	Georg-August-Universität Göttingen, Germany.
H. sapiens: Ohalo II H2	1	N/A	0.06	Tel Aviv University, Israel.
Unknown: StW 311	1	N/A	0.035	Sterkfontein, University of the Witwatersrand, South Africa.
Australopithecus africanus: StW 522	1	N/A	0.04	Sterkfontein, University of the Witwatersrand, South Africa.

Supporting Table 5. Description of landmarks.

**Landmark**	**Description**
1	Medial point on head-neck border at neck midline
2	Lateral point on head-neck border at neck midline
3	Posterior point on head-neck border at neck midline
4	Anterior point on head-neck border at neck midline
5	Superior point at midpoint of the head
6-12	Semi-curve between fixed landmarks 1 and 3
13-19	Semi-curve between fixed landmarks 3 and 2
20-26	Semi-curve between fixed landmarks 2 and 4
27-33	Semi-curve between fixed landmarks 4 and 1
34-241	Surface semilandmarks

References

1. R. Clarke *Australopithecus* from Sterkfontein Caves, South Africa. In: K. Reed, J. Fleagle, R. Leakey (eds) The Paleobiology of Australopithecus. Vertebrate Paleobiology and Paleoanthropology. Springer, Dordrecht (2013).
2. A. I. R. Herries, J. Shaw, Palaeomagnetic analysis of the Sterkfontein palaeocave deposits: implications for the age of the hominin fossils and stone tool industries. *Journal of Human Evolution*, **60**, 523-539 (2011).
3. L. R Berger, J. Hawks, D. J. de Ruiter, S. E. Churchill, P. Schmid, L. K. Delezene, T. L. Kivell, H. M. Garvin, S. A. Williams, J. M. DeSilva, M.M. Skinner, C. M. Musiba, N. Cameron, T. W. Holliday, W. Harcourt-Smith, R. R. Ackermann, M. Bastir, B. Bogin, D. Bolter, J. Brophy, Z. D. Cofran, K. A. Congdon, A. S. Deane, M. Dembo, M. Drapeau, M. C. Elliot, E. M. Feuerriegel, D. Garcia-Martinez, D. J. Green, A. Gurtov, J. D. Irish, A. Kruger, M. F. Laird, D. Marchi, M. R. Meyer, S. Nalla, E. W. Negash, C. M. Orr, D. Radovic, L. Schroeder, J. E. Scott, Z. Throckmorton, M. W. Tocheri, C. VanSickle, C. S. Walker, P. Wei, B. Zipfel, *Homo naledi*, a new species of the genus *Homo* from the Dinaledi Chamber, South Africa. *eLife* 4:e09560 (2015).

4. E. S. Vrba, Chronological and ecological implications of the fossil Bovidae at the Sterkfontein Australopithecine site. *Nature*, **250**, 19-23 (1974).
5. E. S. Vrba, Some evidence of chronology and palaeoecology of Sterkfontein, Swartkrans and Kromdraai from the fossil Bovidae. *Nature*, **254**, 301-304 (1975).
6. E. S. Vrba, 'The significance of bovid remains as indicators of environment and predation patterns', in A. K. Behrensmeyer, A. P. Hill, (eds). *Fossil in the Making*. Chicago: University of Chicago Press, 247-271 (1980).
7. M. Bamford, Pliocene fossil woods from an early hominid cave deposit, Sterkfontein, South Africa. *South African Journal of Science*, **95**, 231-237 (1999).
8. D. M. Avery, The Plio-Pleistocene vegetation and climate of Sterkfontein and Swartkrans, South Africa, based on micromammals. *Journal of Human Evolution*, **41**, 113-132 (2001).
9. M. Sponheimer, J. Lee-Thorp, D. De Ruiter, D. Codron, J. Condron, A. T. Baugh, F. Thackeray, Hominins, sedges, and termites: new carbon isotope data from the Sterkfontein valley and Kruger National Park. *Journal of Human Evolution*, **48**, 301-312 (2005a).
10. M. Sponheimer, D. De Ruiter, J. Lee-Thorp, A. Späth, Sr/Ca and early hominin diets revisited: new data from modern and fossil enamel. *Journal of Human Evolution*, **48**, 147-156 (2005b).
11. K. Kuman, R. J. Clarke, Stratigraphy, artefact industries and hominid associations for Sterkfontein, Member 5. *Journal of Human Evolution*, **38**, 827-847 (2000).
12. K. Kuman, The archaeology of Sterkfontein: preliminary findings on site formation and cultural change. *South African Journal of Science*, **90**, 215-219 (1994a).

13. D. E. Granger, R. J. Gibbon, K. Kuman, R. J. Clarke, L. Bruxelles, M. W. Caffee, New cosmogenic burial ages for Sterkfontein Member 2 *Australopithecus* and Member 5 Oldowan", *Nature,* **522**, 85 (2015).
14. A. I. R. Herries, D. Curnoe, J. W. Adams, A multi-disciplinary seriation of early *Homo* and Paranthropus bearing palaeocaves in southern Africa. *Quaternary Internationa*l, **202**, *14*-28 (2009).
15. R. J. Clarke, The significance of the Swartkrans *Homo* to the *Homo erectus* problem. *Courier Forschungs-Institut Senckenberg* **171,** 85–193 (1994).
16. K. Kuman, The archaeology of Sterkfontein-past and present. *Journal of Human Evolution*, **27**, 471-495 (1994b).
17. E. H. Harmon, The shape of the early hominin proximal femur. *American Journal of Physical Anthropology,* **139**, 154-171 (2009).
18. T. M. Ryan, K. J. Carlson, A. D. Gordon, N. Jablonski, C. N. Shaw, J. T. Stock, Human-like hip joint loading in *Australopithecus africanus* and *Paranthropus robustus*. *Journal of Human Evolution*, **121**, 12-24 (2018).
19. J. K. Mckee, Palaeo-ecology of the Sterkfontein hominids: a review and synthesis. *Palaeontologia Africana*, **28**, 41-51 (1991).
20. K. E. Reed, Early hominid evolution and ecological change through the African Plio-Pleistocene. *Journal of Human Evolution*, **32**, 289-322 (1997).
21. T. R. Pickering, *Taphonomic interpretations of the Sterkfontein early hominid site (Gauteng, South Africa) reconsidered in light of recent evidence*. Ph.D. thesis, University of Wisconsin, Madison (1999).
22. L. C. Bishop, T. R. Pickering, T. Plummer, J. F. Thackeray, Paleoenvironment setting for the Oldowan Industry at Sterkfontein (abstract). Paper for presentation at the XV International Union for Quaternary Research (INQUA), Durban, South Africa (1999).

23. C. J. Luyt, Revisiting palaeoenvironments from the hominid bearing Plio-Pleistocene sites: new isotopic evidence from Sterkfontein. Unpublished MSc thesis, University of Cape Town, Cape Town (2001)
24. C. J. Luyt, J. A. Lee-Thorp, Carbon isotope ratios of Sterkfontein fossils indicate a marked shift to open environments c. 1.7 Myr ago. *South African Journal of Science*, **99**, 271-273 (2003).
25. P. V. Tobias, R. J. Clarke, H. White, 27th Annual Report of the Palaeo-Anthropology Research Unit, University of the Witwatersrand (1993).
26. J. M. DeSilva, K. G. Holt, S. E. Churchill, K. J. Carlson, C. S. Walker, B. Zipfel, L. R. Berger, The lower limb and mechanics of walking in *Australopithecus sediba*. *Science*, **340**, 6129 (2013).
27. D. Marchi, V. S. Sparacello, B. M. Holt, V. Formicola, Biomechanical approach to the reconstruction of activity patterns in Neolithic western Liguria, Italy. *American Journal of Physical Anthropology*, **131**, 447-455 (2006).
28. I. Hershkovitz, M. S. Speirs, D. Frayer, D. Nadel, S. Wish-Baratz, B. Arensburg, Ohalo II H2: A 19,000-year-old skeleton from a water-logged site at the Sea of Galilee, Israel. *American Journal of Physical Anthropology*, **96**, 215-234 (1995).
29. P. Bullough, J. Goodfellow, A.S. Greenwald, J. O'Connor. Incongruent surfaces in the human hip joint. *Nature*, **217**, 1290 (1968).
30. N.Y.P. Afoke, P.D. Byers, W.C. Hutton. The incongruous hip joint. *Journal of Bone and Joint Surgery*, **62-B**, 511-514.
31. W.A. Hodge, R.S. Fijan, K.L. Carlson, R.G. Burgess, W.H. Harris, R.W. Mann. Contact pressures in the human hip joint measured in vivo. *Proceedings of the National Academy of Sciences of the United States of America*, **83**, 2879-2883 (1986).
